# Long-term effectiveness and trajectories of change after treatment with SMART, a transdiagnostic CBT for adolescents with emotional problems

**DOI:** 10.1186/s40359-022-00872-y

**Published:** 2022-07-05

**Authors:** Veronica Lorentzen, Kenneth Fagermo, Bjørn Helge Handegård, Simon-Peter Neumer, Ingunn Skre

**Affiliations:** 1 Department of Psychology, Faculty of Health Sciences, UiT The Arctic University of Norway, 9037 Tromsø, Norway; 2 Department of Child and Adolescent Psychiatry, Divisions of Child and Adolescent Health, University Hospital of North Norway, P.O. Box 19, 9038 Tromsø, Norway; 3 Regional Centre for Child and Youth Mental Health and Child Welfare, UiT The Arctic University of Norway, 9037 Tromsø, Norway; 4 Department of General Psychiatry, University Hospital of North-Norway, P.O. Box 6124, 9291 Tromsø, Norway; 5 Centre for Child and Adolescent Mental Health, Eastern and Southern Norway, 0484 Oslo, Norway

**Keywords:** Cognitive behavioral therapy, Adolescence, Trajectories of change, Emotional disorders, Transdiagnostic, Psychological treatment, Long-term effectiveness, Anxiety, Depression, Child and adolescent mental health services

## Abstract

**Background:**

There is a need for long-term effectiveness trials of transdiagnostic treatments. This study investigates the effectiveness and diagnosis-specific trajectories of change in adolescent patients attending SMART, a 6-week transdiagnostic CBT for anxiety and depression, with 6-month follow-up.

**Methods:**

A randomized controlled trial with waiting list control (WLC) was performed at three child and adolescent mental health outpatient services (CAMHS) in Norway. Referred adolescents (N = 163, age = 15.72, 90.3% females) scoring 6 or more on the emotional disorders subscale of the Strengths and Difficulties Questionnaire (SDQ) were randomly assigned to treatment or to WLC. Long-term follow-up (N = 83, baseline age = 15.57, 94% females) was performed 6 months after treatment completion (Mean = 7.1 months, SD = 2.5). Linear mixed model analysis was used to assess time by group effects in patients with no diagnosis, probable anxiety, depressive disorder, and combined anxiety and depressive disorder.

**Results:**

Almost one third (31%) obtained full recovery according to the inclusion criterium (SDQ emotional). There was highly significant change in all outcome variables. Effect sizes (ES) were largest for general functioning, measured with CGAS (ES: d = 2.19), and on emotional problems measured with SDQ (ES: d = 2.10), while CORE-17, BDI-II and CGAS all obtained ES’s close to 1. There were no significant time by diagnostic group interactions for any outcomes, indicating similar trajectories of change, regardless of diagnostic group. Waiting 6 weeks for treatment had no significant impact on long-term treatment effects.

**Limitations:**

Possible regression to the mean. Attrition from baseline to follow-up.

**Conclusions:**

Six weeks of transdiagnostic treatment for adolescents with emotional problems showed highly significant change in emotional symptoms and functioning at 6-month follow-up. Patients with anxiety, depression, combined anxiety and depression, and emotional problems with no specific diagnoses, all had similar trajectories of change. Hence this transdiagnostic SMART treatment can be recommended for adolescent patients with symptoms within the broad spectrum of emotional problems.

*Trial registration*: ClinicalTrials.gov Identifier: NCT02150265. First registered May 29, 2014.

## Introduction

Anxiety and depression are the most frequent mental disorders in both the general youth population and in those receiving treatment in child and adolescent mental health outpatient services (CAMHS) [1–3]. Anxiety and depressive disorders commonly co-occur during adolescence with rates as high as 75% in clinical samples [4, 5], presenting overlapping symptoms and emotional distress [6]. Untreated, this interferes negatively with numerous mental health outcomes, and can lead to psychological, cognitive, social, and academic impairments [7, 8]. Treatments based on cognitive behavioral therapy (CBT) for both anxiety and depressive disorders have empirical support [9, 10]. Multiple systematic reviews and meta-analyses have reported moderate to large effect sizes (ES) for psychological treatment in youth samples [11–13]. However, the evidence for the effects of these empirically supported treatments rests mainly on efficacy trials. A question raised is to what degree these results hold up when delivered in routine clinical care, as for instance in CAMHS, given the clear differences between the two settings [14–17]. Some have stated that treatments supported by efficacy trials may show reduced treatment effects when transferred to effectiveness trials in CAMHS settings [16, 17]. A recent meta-analysis on CBT for internalizing disorders concludes that CBT delivered in routine care is efficacious in reducing emotional disorders and symptoms, with outcomes comparable to results obtained in efficacy studies. However the authors stated that the quality of the included studies was fair, and the heterogeneity high. Limitations such as varying inclusion criteria of the studies was also highlighted [18]. The participants in this meta-analysis comprised of both children and adolescents, testing various types of CBT, cognitive therapy and behavior therapy. The meta-analysis did not provide information of whether the treatment comprised of transdiagnostic protocols or diagnosis specific treatment protocols. In sum, there are considerable differences between research clinics and routine clinical care. A remaining question is whether short-term transdiagnostic treatment is an effective and lasting treatment for adolescent patients with anxiety and/or depression when delivered in the setting of routine clinical care. Given the high co-occurrence of depression and anxiety in clinical samples, there is a need for effectiveness studies on transdiagnostic treatment that target both anxiety and depression.

As mentioned by Queen et al. [19], youth anxiety and depression share a number of psychological, biological and environmental risk factors (for a review see [20]). Anxiety, depression and traumatic stress have common symptom patterns such as rumination and worry [21], and behavioral avoidance [22]. Moreover, negative affect has been suggested as a latent factor underlying both depressive and anxiety disorders [23, 24]. CBT treatment trials designed to test interventions on single disorders have shown so called “spill-over effects”, where similar response to treatment is shown for comorbid anxiety and depressive disorders [25]. The results of a meta-analysis of CBT for treatment of primary depression are one example demonstrating not only an effect for depressive symptoms, but also “spill-over effects” with reduction in anxiety symptoms [26].

As a consequence of the high comorbidity of emotional disorders among adolescents, and shared vulnerability factor, efforts have been directed towards a transdiagnostic approach to treatment [27–30]. Transdiagnostic treatment is built upon cognitive, behavioral, and physiological processes that are shared or common across diverse disorders. The treatment does not presuppose careful differential diagnostic assessment between disorders belonging to the targeted spectrum, and represents an adoption of an integrative approach [31]. A transdiagnostic intervention for anxiety and depression emerges as an attractive approach for community clinical practice for this target group, offering more flexible interventions compared to standard single-disorder interventions. The use of traditional single-disorder protocols can be time consuming and costly for clinical practitioners [28, 30]. It is hypothesized that transdiagnostic approaches could contribute to lowering the clinical burden in learning several manuals and allow for more flexible interventions to patients presenting with comorbid emotional disorders [27]. As a consequence, a large array of treatment protocols conceptualized as transdiagnostic have been developed and tested. The most extensive research has been performed on the Unified protocol (UP) [32]. The UP exists in many adaptations. Protocol adaptions for children (UP-C; UP Children) and adolescents (UP-A; UP Adolescents) have been developed and tested by Ehrenreich-May and colleagues [33–36] demonstrating promising effects of transdiagnostic treatment for emotional disorders in children and adolescents. There are several additional studies examining various transdiagnostic programs for youth with emotional disorders in different research settings; in primary care [37, 38]; in school settings [39, 40]; and in parent-led teletherapy [41], all showing promising effects. Another promising transdiagnostic therapy protocol for emotional disorders is the Structured Material for Therapy (SMART) [42]. The short-term effectiveness of the SMART treatment was investigated in an RCT with a Norwegian sample of adolescents [43] and small to moderate effect sizes for the time by group interaction effect (ranging from 0.19 to 0.65) were observed for anxiety, emotional symptoms and general functioning, while the effect size for depressive symptoms did not reach significance directly after completion of treatment.

Comparable studies regarding trajectories of change from recruitment, through therapy and to follow-up, are scarce. We found one study employing the UP-A that examined the concurrent trajectories of primary anxiety and depressive symptoms across the course of treatment and at 6 month follow-up. This study showed similar rates of change on self-reported symptoms during the treatment, but whereas anxiety symptoms showed significant improvement after treatment, depressive symptoms seemed to plateau [19]. Investigating effects of transdiagnostic treatment provides the opportunity to examine whether there are separate trajectories of change when it comes to depression, anxiety, and comorbid anxiety and depression for patients in a CAMHS setting, in order to show whether they present similar or different rates of change both post-treatment and at follow-up.

Most studies measure post-treatment effects, but less frequently incorporate follow-up measurements conducted months after the end of treatment. Despite the strong evidence for youth CBT post-treatment effectiveness, relapse after treatment can be observed in as many as one third to one half of treated youths (e.g. [44, 45]). So far, our knowledge about long-term effects of CBT for emotional disorders is limited. A meta-analysis performed by Rith-Najarian and colleagues [46] provides support for stability of treatment effects of CBT for youths in a long-term follow-up with rather large within-subject effect sizes (g = 1.23–1.82), but also highlights the need for several improvements in research standards, with an emphasis on prioritizing assessment at long-term follow-up. Many studies conduct limited follow-up assessments only 2–3 months post-treatment (e.g. [11, 13]). Longer follow-up studies are important for several reasons and are needed to understand the persistence of treatment effects of CBTs [46]. A meta-analysis on depression demonstrated that treatment effect sizes were negatively correlated with duration from end of treatment until follow-up [26]. The same study found that treatment duration was not correlated with outcome, suggesting that some briefer treatments may have potential to be as effective as lengthier ones [26]. Number of sessions or weeks of treatment did not moderate the effect size or remission rates in a recent meta-analysis investigating CBT for children and adolescents treated for internalizing disorders in routine clinical care [18]. This could indicate that shorter treatments could be as effective as lengthier ones.

In addition to duration of treatment, there are other issues worth examining, such as effectiveness in adolescent samples and the effects of waiting for treatment. In general, there is a need for more research on adolescents, especially since we know that emotional disorders persist if left untreated [47]. A meta-analysis examining effectiveness of anxiety treatment in children and adolescent, has shown that the research is mainly based on children, despite the potential strong effects shown by the limited number of studies on adolescents [48].

A relevant question for clinical trials with a waiting list control group, is to study the effect of waiting time before receiving treatment. Waiting time before commencing treatment is frequently used as a quality benchmark for health services, also for Norwegian CAMHS. For somatic illness and for mental health conditions with high risk for harm or self-harm, waiting time can obviously imply risk of deterioration or a fatal outcome. However, little is known about whether other young patients react negatively to waiting, for instance by reduced attendance, or by developing a more treatment resistant condition while waiting [49]. Will the delayed onset of therapy after enrollment influence the short- and long-term outcome of therapy? This question has clinical implications, since most patients in CAMHS wait before starting treatment.

### Aims

The aim of the present study was to examine the long-term effectiveness, and diagnostic group specific change trajectories of a six-session transdiagnostic CBT for young patients (age 14–17) with depression, anxiety and combined anxiety and depression, treated in regular CAMHS, by

1. Examining treatment effects at follow-up 6 months after treatment completion.
2. Examining the impact of waiting 6 weeks before start of therapy on the long-term treatment effect.
3. Examining change trajectories for patients with diagnoses of pure anxiety, pure depression, and combined anxiety and depression, from pre-treatment, through treatment, and at 6-month follow-up.

## Methods

This is a 6-month follow-up study of adolescent patients participating in a randomized controlled clinical trial with waiting list control. The active treatment was transdiagnostic CBT, according to the treatment manual Structured Material for Therapy (SMART) [42], delivered by the clinical staff at community child and adolescent mental health service clinics (CAMHS). The pre- to post-treatment short-term evaluation of the SMART treatment protocol for the present study sample is described in Lorentzen et al. [43], including more detailed information about the SMART treatment protocol, clinical setting, training of therapists, randomization procedure, treatment integrity, user satisfaction, and therapeutic alliance.

### Procedure

**Fig. 1:**
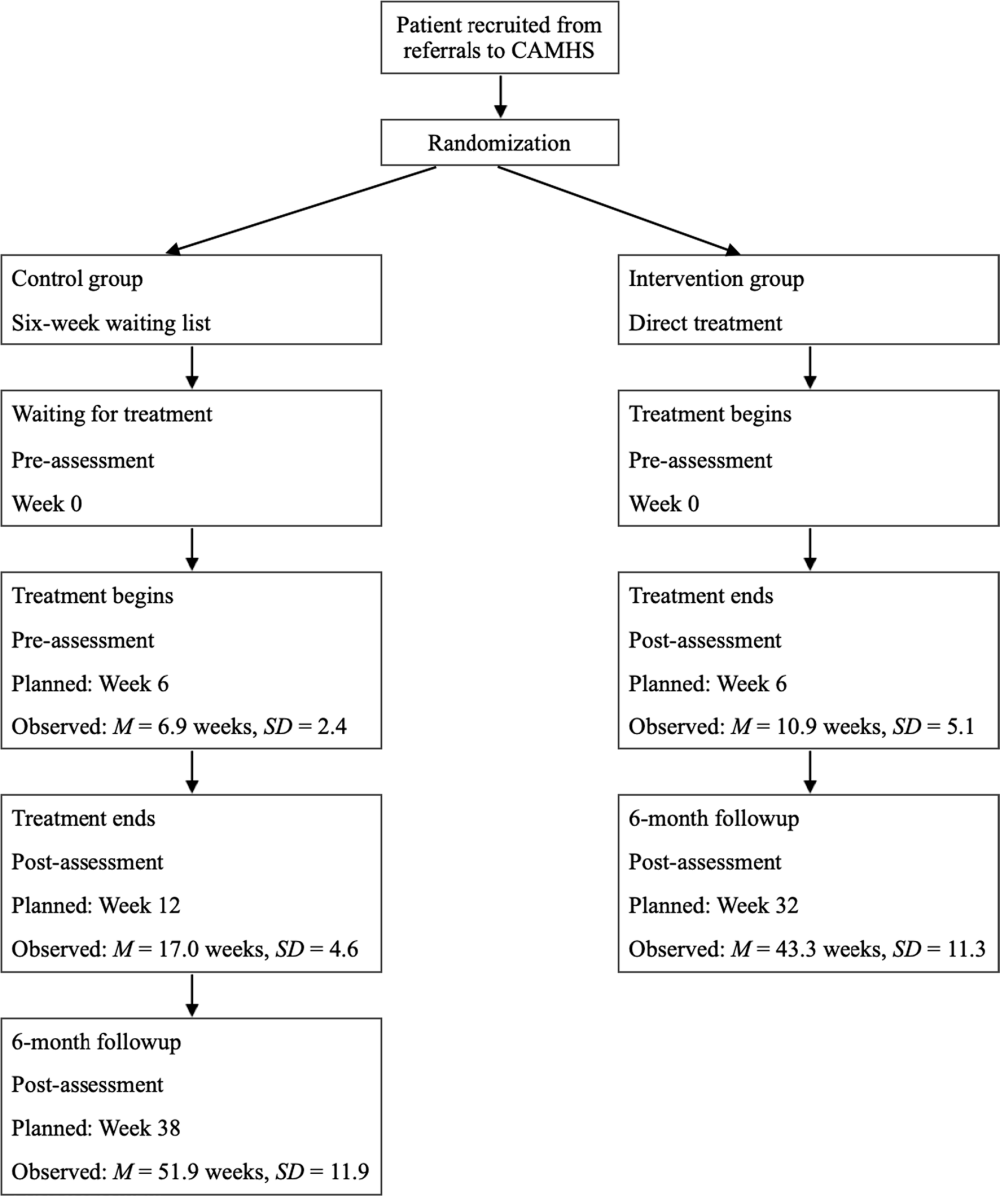
Flowchart showing points of, and time between measurements in weeks

**Fig. 2:**
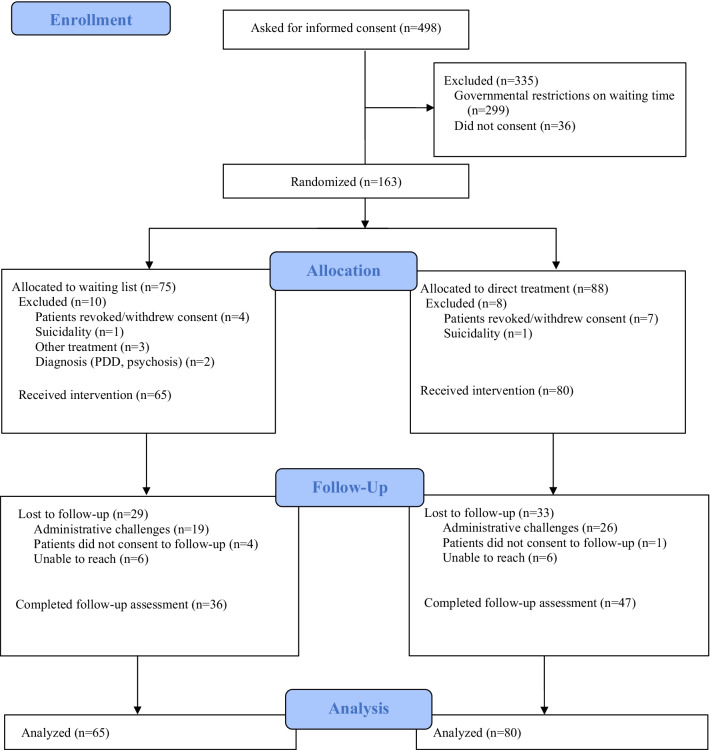
Consort flow diagram

We asked a total of 498 patients referred to CHAMS for informed consent, from January 2012 to June 2016. Of these, 335 were excluded due to governmental restriction on waiting time prior to start of treatment. Patients in the direct intervention group were pre-evaluated and commenced treatment with SMART immediately after enrollment. After completion of the 6-week SMART treatment, they were evaluated post-treatment. The patients in the waiting list control (WLC) condition were pre-evaluated twice; first at enrollment, and secondly after a 6-week waiting period, prior to commencing treatment with SMART. The WLC group went through post-treatment evaluation immediately after completing the 6-week SMART treatment, 12 weeks after enrollment. Follow-up was scheduled 6 months post-treatment. Figure 1 shows timepoints for measurements in weeks. Figure 2 shows the consort flow diagram.

The mean duration of time between post-treatment evaluation and follow-up was 32.8 weeks (SD = 10.8, range 28–90).

### Participants

The participants are described in Lorentzen et al. [43]. The full sample included 145 adolescents (aged 14–17, *M* = 15.72, *SD* = 1.14, 90.3% females), recruited from referrals to three public child and adolescent mental health outpatient clinics, from January 2012 to June 2016. During the routine intake procedure at the clinics, the participants who fulfilled the inclusion criteria described below were informed about the study and asked to participate. Adolescents over 16 years and parents of children under 16 years signed informed consent. Inclusion criteria were (a) age between 14 and 17 years; (b) a score of at least 6 on the Strength and Difficulties Questionnaire (SDQ) screening tool emotional scale; and (c) maintenance of a maximum waiting time for necessary medical care of 6 weeks given by Norwegian health authorities. Exclusion criteria were (a) a diagnosis of pervasive developmental disorder (PDD); (b) psychotic symptoms; (c) use of anxiolytic or anti-depressant medication during the treatment period; and (d) patients who did not speak the Norwegian language. A total of 199 adolescents were assessed for eligibility and were asked for informed consent. Of these, 36 did not consent. A total of 163 were randomized to either treatment or waiting list. In the current study, a total of 18 patients did not complete the treatment or waiting phase. Of these, we had no information for the reason for non-completion for 11 patients, 2 cited lack of motivation, 3 were referred to other treatment (2 received trauma treatment, 1 regular cognitive behavioral therapy), and finally 3 withdrew because of geographical distance (2 moved to another location, 1 had a long distance to travel to get to the CAMHS). The main administrative challenge to completing the follow-up was difficulty getting into contact with the patients, and in some cases the therapists forgot to perform follow-up after 6 months. Results for the final study sample are presented according to the CONSORT flow diagram in Fig. 2.

### Treatment

The adolescents were treated with the SMART program [43]. The SMART program is a transdiagnostic CBT program with materials organized in five modules (introduction, depression, anxiety, assertiveness training, and summary, in a total of eight sessions). In this study, the core modules for anxiety and depression, except the module for assertiveness training (2 sessions), were employed as a standard brief therapy in the outpatient clinics. Norwegian law restricts waiting time in CAMHS, and by excluding assertiveness training both groups had similar time intervals (6 weeks waiting time and 6 weeks treatment). Four modules were given over 6 sessions, each with a duration of 90 min.

The program has proven its initial effectiveness [43] and is based on well supported methods for treating anxiety and depression with emphasis on cognitive restructuring, exposure and activation. The modules consist of a definition of individual treatment goals, activation of personal resources, behavioral experiments, information about emotional problems and related coping strategies. Although the treatment is modular and flexible, it was delivered in a linear manner for research purposes.

### Measures

The following measures were employed in the present study:

#### Diagnostic assessment: DAWBA

*Development and Well Being Assessment (DAWBA)* [50] is a multi-informant computer-administered diagnostic interview, with both open- and closed-ended questions. It is administered at intake as a digital self-report instrument in the participating CAMHS. The data is gathered through the authorized DAWBA online system and published in accordance to the copyright terms and with permission from the copyright holder. In the present study, only information from the patients was used. DAWBA uses computer algorithms to suggest the likelihood of diagnoses, ordering the probability of a diagnosis into DAWBA bands ranging from 0 to 5 [51]. The top two bands (4 and 5) suggest a ≈ 50% and > 70% likelihood that the patient meets criteria for the disorder. Goodman and colleagues [50] found that DAWBA could discriminate between community and clinic samples of youth, and later [51] found that DAWBA is well suited to find approximate prevalence of disorders. When comparing the computer algorithms to clinician rated diagnoses, Goodman et al. [51] found that DAWBA can underestimate the prevalence of disorders on a group level. On an individual level, kappa values showed agreement between 0.4 and 0.7, specificity 0.98–0.99, positive predictive values 0.5–0.8 and negative predictive values 0.96–0.99.

#### Inclusion and main outcome measure for the present study: SDQ

*The Strengths and Difficulties Questionnaire (SDQ)* [52] was administered as a part of the authorized DAWBA online system. Data is published in accordance with the copyright terms and with permission from the copyright holder. SDQ was administered at enrollment, end of therapy and at 6 month follow-up. The main inclusion criteria and the primary outcome measure for emotional symptoms was the emotional problems subscale on the self-rated SDQ for 11–17 year-olds. SDQ is a brief emotional and behaviour screening questionnaire where symptom items are scored on a 3-point Likert scale, from 0 (not true) to 1 (somewhat true) to 2 (certainly true). The maximum score on the emotional subscale is 10, and based on a Norwegian sample [53] we used a cut-off of 6 or above to separate a clinical from a non-clinical population. SDQ is a frequently used screening instrument and has satisfactory psychometric properties [54, 55]. In this study, we used only the emotional symptoms subscale, which has shown acceptable reliability and adequate internal consistency [54]. Internal consistency in our sample was acceptable for the SDQ emotional scale (Cronbach’s *α* = 0.70).

#### General functioning: CGAS

*The Children's Global Assessment Scale (CGAS)* [56] was used as a secondary outcome measure for general level of function, and is a routine tool employed in the participating CAMHS. CGAS is a therapist-scored numeric scale ranging from 1 to 100, with high scores indicating a higher level of functioning and a score lower than 70 as the clinical cut-off point. In this study a group of at least 3 experienced clinicians scored each child’s clinical profile blindly, and the scores were averaged. The clinicians had used CGAS routinely in clinical practice, and had extensive experience with the instrument. CGAS has shown good psychometric properties [57]. In the present sample, there was high inter-rater reliability between the three CGAS raters (ICC = 0.97).

#### General psychological distress: CORE-OM and CORE-17

*Clinical Outcome in Routine Evaluation-Outcome Measure (CORE-OM)* [58] was used as a secondary outcome measure for general psychological distress, and risk of suicide and self-harm, and was introduced for the purposes of the present study. CORE-OM, developed in the UK [47], is widely used as a general psychotherapy and consulting outcome measure in mental health outpatient and consulting services [59–61], and has been translated into 20 languages [62–65]. Originally CORE-OM is a 34-item questionnaire with items using a 5-point Likert scale, giving an average score between 0 and 4, where high scores indicate an increased symptom severity. CORE-OM includes items related to well-being, anxiety, depression, trauma reactions, sleep, bodily pain, daily functioning and risk of harm to self and others. CORE-OM has shown good psychometric properties [58, 62], but also has some methodological challenges [60]. In the present study a 17-item general problem scale score was used, based on the results of a validation of the CORE-OM in Norwegian adolescents; a community sample from junior and senior high-school, and the clinical sample in the present study [66]. The CORE General problem scale score reports symptoms and problems, both psychologically and in relation to others, and can be interpreted as a measure of general psychological distress [66]. In this study, the clinical cut-off point for CORE-17 was set at 1.3, based on the mentioned validation study [66]. The internal consistency of the CORE-17 subscale was excellent (Cronbach’s *α* = 0.90).

#### Depressive symptoms: BDI-II

*Beck Depression Inventory, second edition (BDI-II)* [67] was used as a secondary outcome measure for extent and depth of depressive symptoms. BDI-II is a frequently used instrument in the CAMHS, but is not a mandatory tool. BDI-II is a 21-item questionnaire with items using a 4-point Likert scale, giving a maximum score of 63. Cut-off scores suggested by Beck et al. [67] were between 14 and 19 for mild depression, 20–28 for moderate depression and 29–63 for severe depression. BDI-II has shown good psychometric properties [68–70]. The internal consistency for the BDI-II in the present sample was excellent (Cronbach’s *α* = 0.91).

#### Anxiety symptoms: MASC

*Multidimensional Anxiety Scale for Children (MASC)* [71] was used as a secondary outcome measure for the degree of anxiety. In this study we used the MASC total score. MASC is a 39-item questionnaire with items scored on a 4-point Likert scale. High scores indicate a higher degree of anxiety. MASC has shown good psychometric properties [71, 72]. The internal consistencies of the MASC subscale scores in the sample varied (Cronbach’s *α* between 0.57 and 0.80).

### Data analysis

This study was part of a randomized controlled effectiveness trial of transdiagnostic CBT for adolescents. The power calculations have been reported in Lorentzen et al. [43], and therefore, will not be presented in detail here.

The reliable change index (RCI) [73] was used as an evaluation of clinically significant and/or reliable change. RCI was evaluated on the main inclusion and outcome measure; the SDQ emotional scale. The inclusion criterium in this study was a score of at least 6 on the SDQ emotional scale, and hence, the criterium for clinically significant change was a score lower than 6. The criterium for reliable change, was that the magnitude of change was statistically significant, and in the present study reliable change was calculated to be a change of at least 4 scale points on the SDQ.

To test the time by group and time effects, we used linear mixed model analysis [74]; measurement occasion (level 1; pre, post and follow-up) is nested within individuals (level 2). In this analysis, time was treated as a continuous variable, and represented as the number of days since baseline for the different measurement occasions. A random slope model was used. We computed estimated marginal means and standard errors in a linear mixed model analysis where time was treated as categorical, in order to get model-based predictions for each treatment condition or diagnostic group on each measurement occasion.

Effect sizes for the time by group effect and the time effect were computed as the unstandardized coefficient (computed by the LMM analysis) divided by the pooled within-group standard deviation at baseline. This ratio was multiplied by the average number of days from baseline to the follow-up measurement in the total sample, since the unstandardized coefficient was given in change difference per day or change per day [75]. Follow-up was performed after on average 327 days since the baseline measurement (minimum = 151, maximum = 632).

For handling of pretreatment differences between the groups and dropout, see Lorentzen et al. [43].

We used IBM SPSS v25 for all analyses, and 0.05 was used as significance level.

## Results

### Additional treatment after completing SMART

Patients who were in need of further treatment after the SMART intervention, were taken care of in routine care. Data obtained from the CAMHS case records show that after completing SMART (post-treatment) and before the 6-month follow-up, 33 (22.8%) patients had zero additional sessions, 22 (15.2%) had 1 additional session, 7 (4.8%) had 2 additional sessions, 9 (6.2%) had 3 additional sessions and 62 (42.8%) had 4 or more additional sessions after the SMART intervention. We had no information about additional treatment for 12 (8.3%) patients after they received the SMART intervention. We previously analyzed the association between the number of sessions of treatment and change in general functioning and mental health from post-treatment to follow-up. We found no time by additional treatment interaction on any of the dependent variables (no association between the number of sessions of extra treatment and change in the dependent variables from post-treatment to follow-up), and therefore did not include the additional treatment variable in models used for analysis of long-term effects of treatment or diagnostic groups.

Table 1 shows diagnoses and comorbidity, and the resulting distribution of the patients into direct treatment or WLC group. More than half (n = 80 (55%)) had a probable diagnosis of one or more anxiety disorder(s), and generalized anxiety disorder and social phobia were the most frequent probable anxiety diagnoses. More than half the participants (n = 75 (52%)) had a probable diagnosis of depression. Nearly a quarter of the participants had a probable pure anxiety disorder, one fifth had a probable pure depressive disorder, and nearly one third had a probable diagnosis of both anxiety and depression. A quarter of the participants did not have probable diagnosis of either an anxiety or a depressive disorder. There were too few males in the sample to perform gender comparisons. The randomization was performed independently of the diagnostic assessment. However, the two groups had fairly equal distributions of diagnoses.

**Table 1:** Diagnoses (n = 145)

DAWBA prediction	Total		WLC		Direct treatment	
	n	% (of 145)	n	% (of 65)	n	% (of 80)
Pure anxiety	34	23.4	18	27.7	16	20.0
Pure depression	29	20.0	12	18.5	17	21.3
Depression and anxiety	46	31.7	18	27.7	28	35.0
Depression and GAD	30	20.7	10	15.4	20	25.0
Depression and Social phobia	27	18.6	10	15.4	17	21.3
Depression and specific phobia	7	4.8	0	0.0	7	8.8
Depression and agoraphobia	9	6.2	4	6.2	5	6.3
Depression and panic disorder	6	4.1	4	6.2	2	2.5
No diagnosis of anxiety or depression	36	24.8	17	26.2	19	23.8

Diagnoses in both ICD-10 and DSM-IV (same algorithm)

### Clinically significant and reliable change from baseline to follow-up

The rates of clinically significant and/or reliable change on the main inclusion criterium of emotional symptoms from the SDQ is presented in Table 2.

**Table 2:** Clinically significant and reliable change on the SDQ emotional scale from baseline to follow-up

	Reliable change^b^	
	No	Yes
*No clinically significant change*		
n	26	4
% of Total	44.8%	6.9%
*Clinically significant change*^a^		
n	10	18
% of Total	17.2%	31.0%

^a^Clinically significant change: The number of patients with SDQ emotional score < 6 at follow-up

^b^Reliable change: The number of patients with a change of at least 4 scale points on the SDQ emotional scale from baseline to follow-up

Nearly half the patients reported clinically significant change on the SDQ emotional scale from baseline to follow-up, indicating that at follow-up they were scoring below the clinical cut-off point, and hence had a subclinical score on this instrument. Close to 40% of the patients showed statistically significant change from baseline to follow-up, indicating reliable change. Nearly a third of the patients fulfilled the criteria for both, and thus showed clinically significant and reliable change in scores from baseline to follow-up on the SDQ emotional scale.

Looking at change in general functioning from baseline to follow-up in the present sample, only 1 (0.09%) out of 109 patients were rated below the clinical cut-off point on CGAS at baseline, while nearly half the patients; 29 out of 64 (45.3%), had a CGAS score above the clinical cut-off point at follow-up.

### Group differences in change and overall effect sizes from baseline to follow-up

Table 3 reports effects of time from baseline to follow-up for the outcome variables in the overall sample. There was a highly significant change in the sample for all outcome variables, and the change was in the hypothesized direction. Effect sizes were largest for general functioning, where the predicted change at follow-up corresponded to 2.19 standard deviations increase in the CGAS score, and for the SDQ emotional problems scale there was a predicted decrease equal to 2.10 standard deviations. For the secondary outcome measures of depressive symptoms (BDI-II), anxiety symptoms (MASC) and general psychological distress (CORE-17), effect sizes corresponded to a decrease of approximately 1 standard deviation.

**Table 3:** Estimated marginal means (and standard errors) for measures on functioning, emotional problems, anxiety and depression

Measure	Treatment			Wait-list				Time* group F	Time*group effect size at T3 *d* = b*327/*SD*^ab^	Overall change effect size *d* = b*327/*SD*^b^
	Pre-treatment start 0 weeks	Post-treatment ends 6 weeks	6 months follow-up	Pre-waiting for treatment	Pre-treatment start 6 weeks	Post-treatment ends 12 weeks	6 months follow-up			
CGAS	51.77 (1.40)	61.57 (1.47)	69.00 (1.72)	49.03 (1.65)	52.13 (1.52)	59.33 (1.63)	64.78 (2.03)	0.15	− 0.19	F = 83.4**/d = 2.19
BDI total	28.98 (1.43)	19.74 (1.50)	15.59 (1.78)	29.60 (1.61)	24.12 (1.60)	18.68 (1.71)	15.76 (1.94)	0.77	0.21	F = 74.3**/d = − 1.02
MASC total	60.54 (2.02)	50.42 (2.11)	45.89 (2.53)	61.29 (2.27)	57.61 (2.26)	50.01 (2.39)	46.26 (2.73)	0.15	0.10	F = 47.0**/d = − 0.91
CORE 17	2.25 (0.09)	1.66 (0.10)	1.46 (0.11)	2.43 (0.10)	2.03 (0.10)	1.65 (0.11)	1.50 (0.12)	0.03	− 0.02	F = 60.2**/d = − 1.19
SDQ emotion	7.90 (0.22)	6.54 (0.25)	4.90 (0.33)	8.11 (0.27)	7.30 (0.26)	6.20 (0.29)	5.96 (0.37)	3.90	1.03	F = 59.0**/d = − 2.10

*< 0.05, **p < 0.0005

^ab^Unstandardized coefficient for the time by group effect (change difference per day), and SD is the pooled within-group standard deviation at baseline

^b^Measured on average 327 days from baseline

The patients in the two treatment conditions received the SMART treatment over different time schedules. The adolescents in the treatment group started treatment immediately after baseline, while the waiting list condition waited 6 weeks before initiation of the treatment. Apart from this 6-week waiting time, the conditions for the two groups were equal. To test whether the groups differed in change from baseline to follow-up, i.e., to test whether the time schedule affected overall change rates, linear mixed models’ analyses were performed. Table 3 shows results from these linear mixed model analyses for the five outcome variables.

There were no statistically significant time by group interactions for any of the outcomes [Self-reported emotional problems (SDQ); General functioning (CGAS); Anxiety (MASC); Depression (BDI-II); General psychological distress (CORE-17)]. The effects computed at follow-up were small, except for SDQ emotional problems, where the difference in change rates at follow-up corresponded to a standardized effect size of approximately 1.0 standard deviations. These results indicate that there was not sufficient evidence for different change rates in the two experimental conditions from baseline to follow-up.

### Trajectories of change according to diagnostic groups

**Fig. 3:**
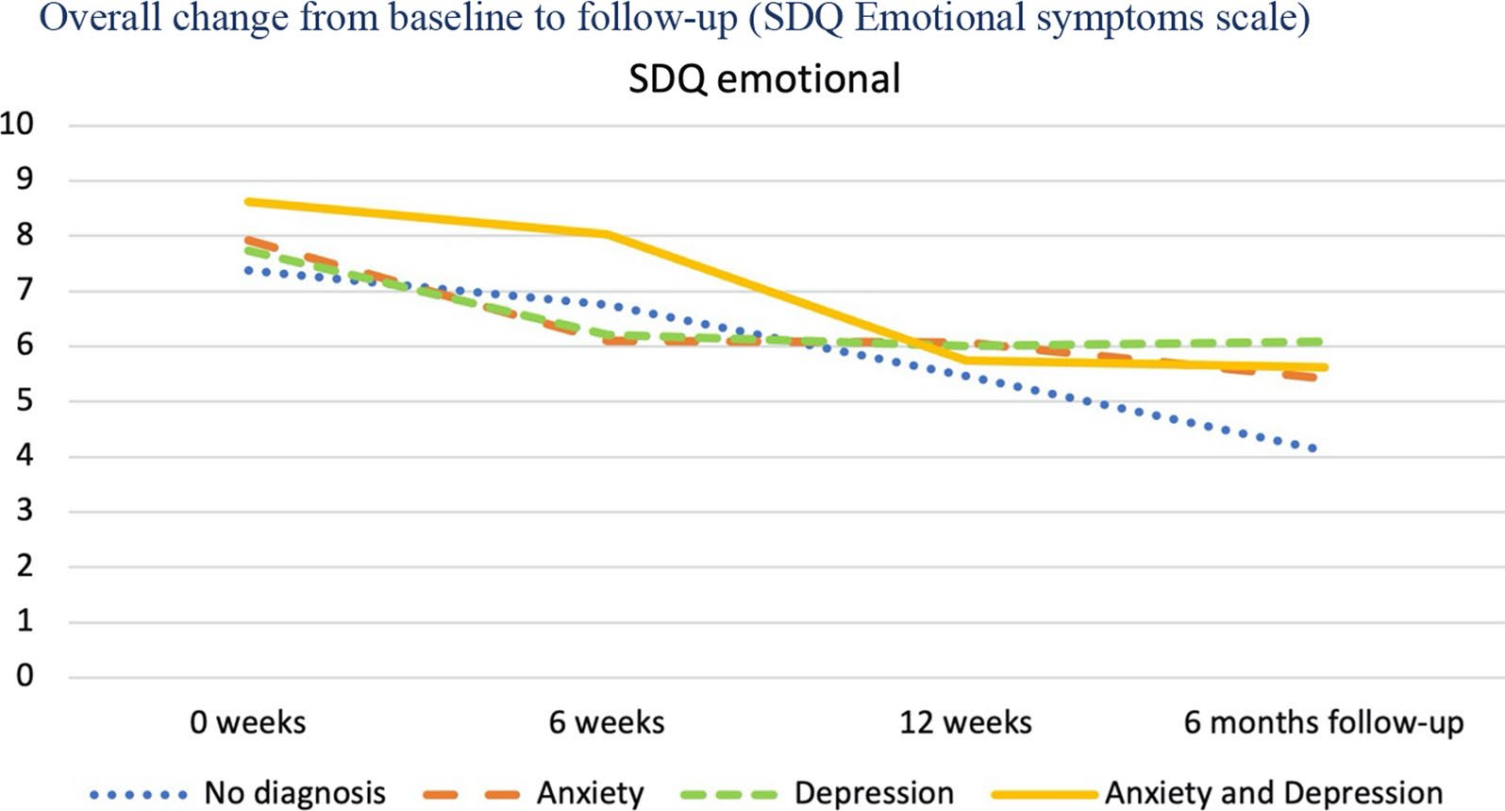
Overall change from baseline to follow-up (SDQ Emotional)

**Fig. 4:**
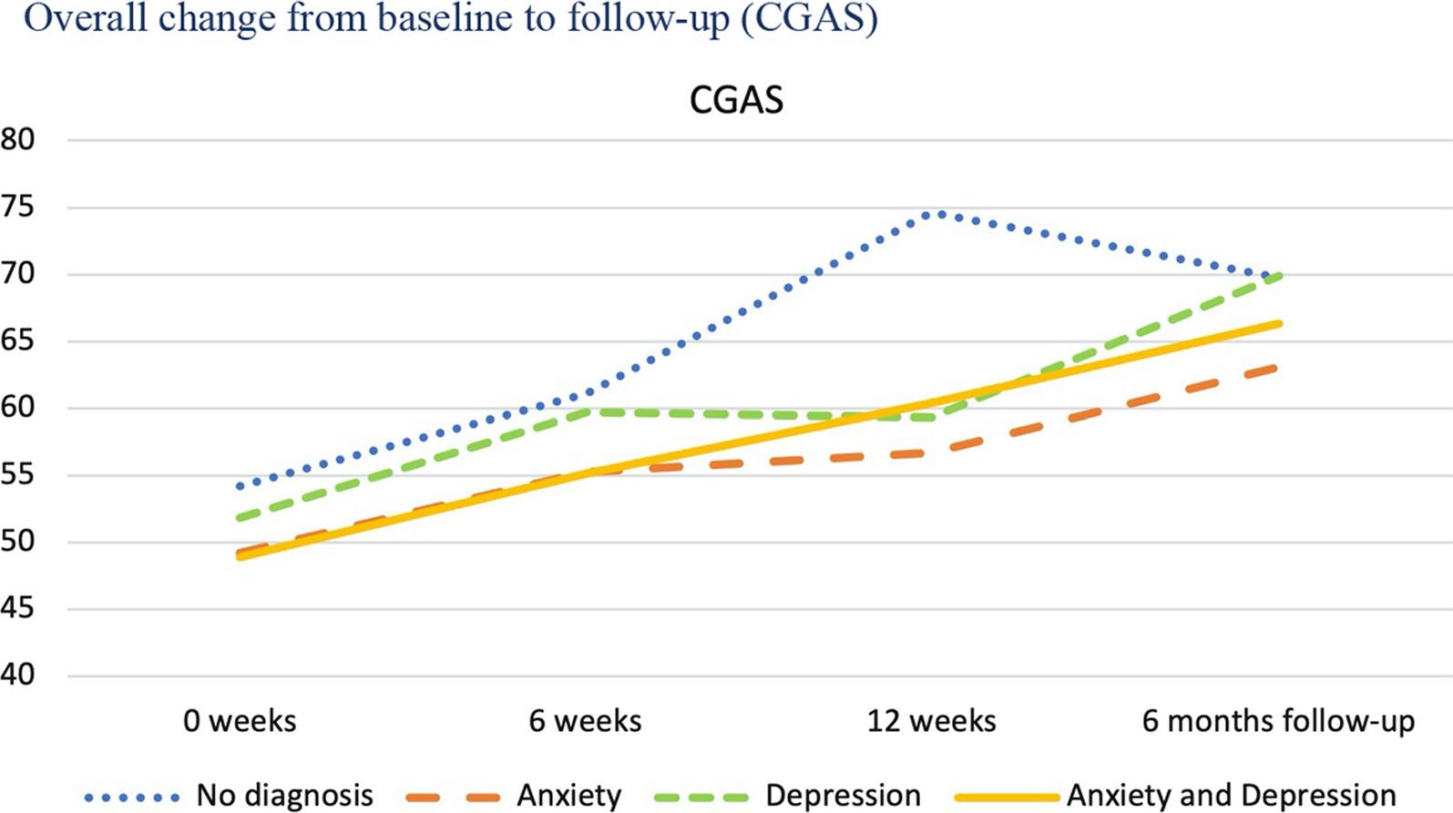
Overall change from baseline to follow-up (CGAS)

**Fig. 5:**
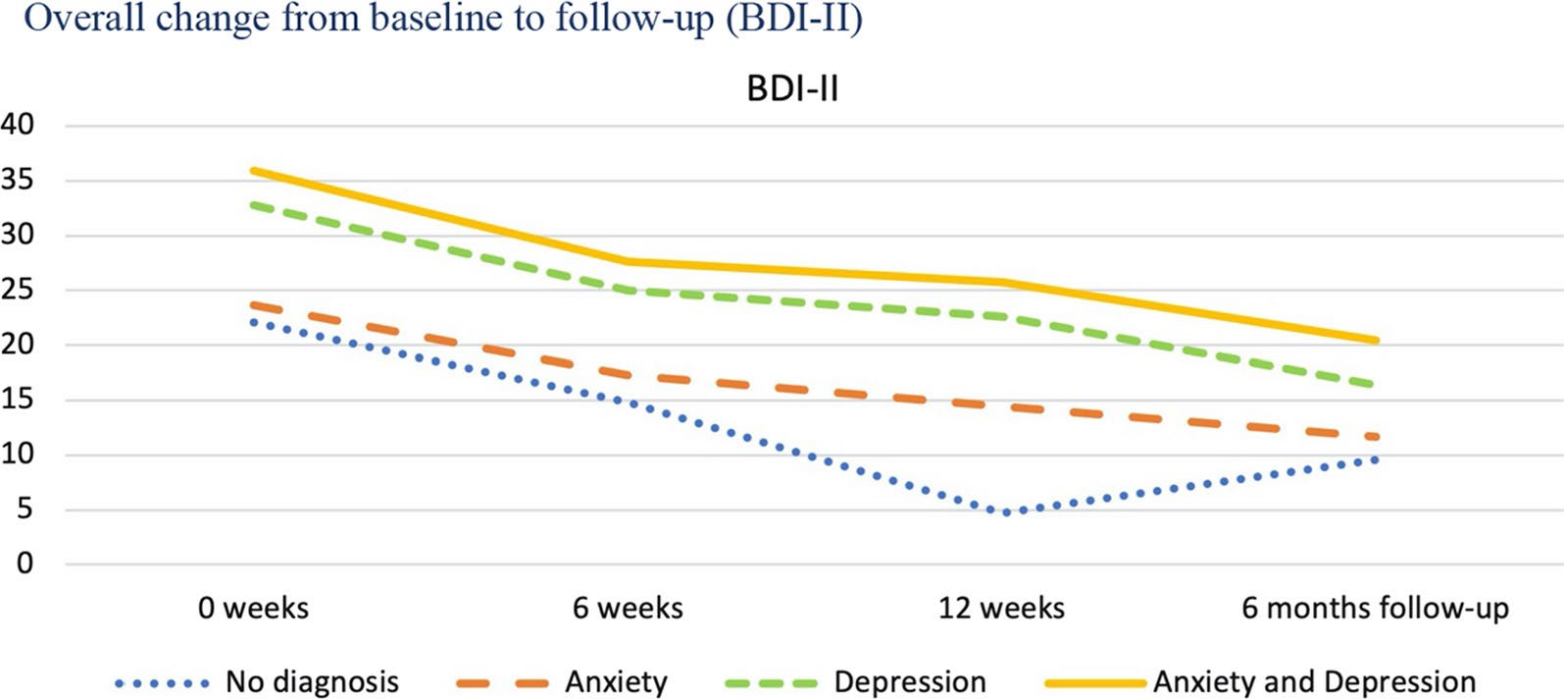
Overall change from baseline to follow-up (BDI-II)

**Fig. 6:**
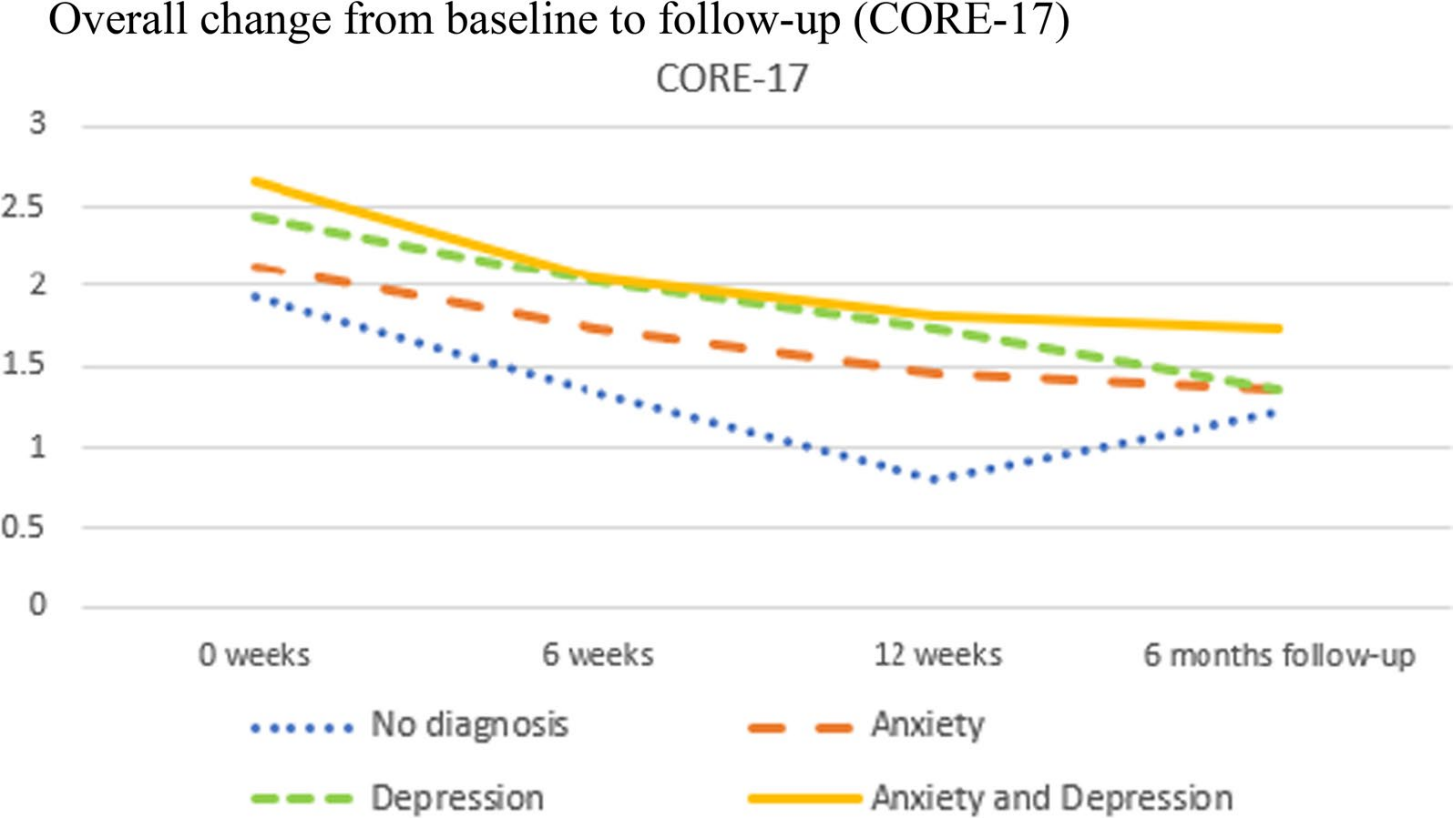
Overall change from baseline to follow-up (CORE 17)

**Fig. 7:**
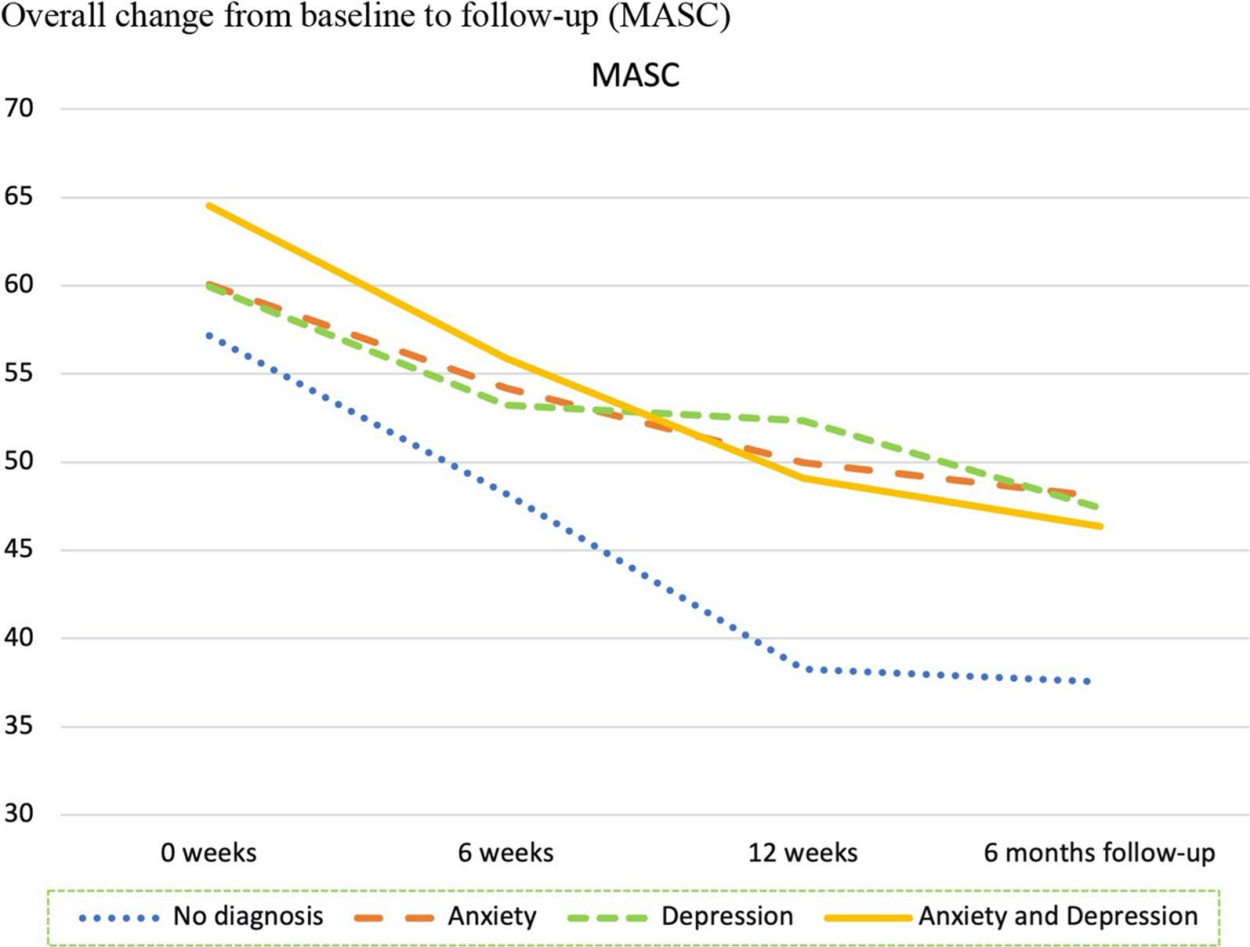
Overall change from baseline to follow-up (MASC)

As previously shown in Table 1, each individual was classified into one of four diagnostic categories based on the DAWBA prediction levels 4–5, corresponding to at least 50% probability of the disorder. The four diagnostic categories were: No probable anxiety or depression; pure anxiety; pure depression; both anxiety and depression. We then investigated the trajectories of change for these four diagnostic groups and tested whether they changed differently on the five primary and secondary outcome measures from baseline to follow-up. The results are presented in Table 4 for overall results, Fig. 3 for SDQ emotional scale, Fig. 4 for CGAS, Fig. 5 BDI-II, Fig. 6 for CORE-17, Fig. 7 for MASC.

**Table 4:** Estimated marginal means (standard errors) at four measurement occasions for four diagnostic groups

Scale	Diagnose group	0 weeks	6 weeks	12 weeks	Follow-up	F^a^
		EMM (SE)	EMM (SE)	EMM (SE)	EMM (SE)	t*g
CGAS	No diagnosis of anxiety or depression	54.23 (2.49)	61.11 (2.49)	74.64 (4.14)	69.77 (3.14)	0.19 NS
	Pure anxiety	49.23 (2.35)	55.28 (2.27)	56.70 (2.89)	63.10 (3.10)	
	Pure depression	51.85 (2.48)	59.72 (2.44)	59.34 (3.60)	69.90 (2.59)	
	Anxiety and depression	48.91 (1.81)	55.15 (1.83)	60.42 (3.14)	66.31 (2.24)	
BDI-II	No diagnosis of anxiety or depression	22.07 (2.25)	14.83 (2.38)	4.71 (4.02)	9.59 (2.93)	0.23 NS
	Pure anxiety	23.66 (2.06)	17.29 (2.11)	14.47 (2.78)	11.68 (2.78)	
	Pure depression	32.75 (2.26)	24.96 (2.24)	22.64 (3.22)	16.36 (2.68)	
	Anxiety and depression	35.94 (1.79)	27.63 (1.85)	25.74 (2.98)	20.40 (2.19)	
MASC	No diagnosis of anxiety or depression	57.15 (3.43)	48.21 (3.62)	38.26 (5.57)	37.54 (4.53)	0.87 NS
	Pure anxiety	60.06 (3.15)	54.18 (3.22)	49.97 (4.16)	48.03 (4.16)	
	Pure depression	59.95 (3.45)	53.24 (3.41)	52.34 (4.80)	47.38 (4.04)	
	Anxiety and depression	64.54 (2.74)	55.89 (2.81)	49.09 (4.43)	46.35 (3.30)	
CORE-17	No diagnosis of anxiety or depression	1.94 (0.15)	1.34 (0.16)	0.80 (0.26)	1.21 (0.20)	0.86 NS
	Pure anxiety	2.13 (0.14)	1.74 (0.14)	1.46 (0.19)	1.35 (0.18)	
	Pure depression	2.44 (0.15)	2.03 (0.15)	1.74 (0.22)	1.36 (0.17)	
	Anxiety and depression	2.66 (0.12)	2.05 (0.12)	1.81 (0.20)	1.73 (0.14)	
SDQ emotional symptoms	No diagnosis of anxiety or depression	7.37 (0.36)	6.75 (0.42)	5.46 (0.68)	4.11 (0.54)	1.44 NS
	Pure anxiety	7.91 (0.33)	6.10 (0.35)	6.07 (0.50)	5.42 (0.50)	
	Pure depression	7.73 (0.36)	6.21 (0.38)	6.01 (0.63)	6.08 (0.50)	
	Anxiety and depression	8.61 (0.29)	8.03 (0.32)	5.74 (0.63)	5.62 (0.43)	

*NS* not significant, *EMM* estimated marginal means

^a^F-test for time by diagnostic group interaction, where time is treated as a continuous variable (days since baseline)

Table 4 and Figs. 3, 4, 5, 6 and 7 shows no significant time by diagnostic group interactions on any of the outcome measures, indicating similar patterns of change in the four diagnosis groups. All diagnostic groups had change in the clinically desired direction on all measures from baseline to follow-up. The patient group with no probable diagnosis of anxiety or depression, had a more fluctuating trajectory than the other diagnostic groups, but this group was also the smallest, and thus we believe these fluctuations may have happened by chance, due to the small sample size. Furthermore, all three groups with emotional disorders had similar change trajectories; and all had the same change profile from baseline, with a break point post-intervention, and a steady slope to follow-up, with a continuing change in the clinically desired direction. Albeit that there were no statistically significant differences between the trajectories, it is interesting to note that the comorbid anxiety and depression group had more severe scores at almost all measurement occasions and on all outcome variables.

## Discussion

The present study investigated the long-term effectiveness and trajectories of change for adolescent patients treated with the transdiagnostic CBT program SMART in a CAMHS setting. The main findings at 6-month follow-up was, firstly, that 6 weeks of transdiagnostic treatment for emotional problems yielded recovery in nearly half the patients measured at follow-up, and clinically significant and reliable change in nearly one third, according to the main inclusion criterium SDQ emotional symptoms. Secondly, nearly half the patients were rated as having normal functioning on the outcome measure CGAS at follow-up. The analysis from baseline to follow-up showed a highly significant change in the overall sample for all outcome variables (emotional symptoms, functioning, general psychological distress, depressive and anxiety symptoms), and the effect sizes were all well above what Cohen characterized as large effects [76]. However, it is important to note that these effects sizes were within-effect sizes and can only be denoted as large when compared to other within-effect sizes. Furthermore, the change trajectories for the young patients receiving this transdiagnostic treatment, were similar for pure anxiety, pure depression, anxiety and depression combined, and patients with emotional problems without a specific diagnosis. Finally, our findings indicate that waiting 6 weeks before commencing treatment with the SMART program, seemed to have had no influence on the long-term outcome for these adolescent patients.

The magnitude of the observed changes indicates a significant drop in emotional symptoms, both anxiety and depression, and psychological distress, on all measures, and a heightening of psychological functioning. Compared to the changes observed at post-treatment, immediately after conclusion of the 6-week SMART program [26], the long-term effectiveness was even more pronounced, and the effect sizes were statistically significant on all outcome measures. Thus, the improvement seems to have continued from post-treatment to follow-up.

There are few comparable effectiveness studies with similar participants, clinical settings, treatment duration, and transdiagnostic treatment, but these changes are in line with those found in a meta-analysis of durability of effects of treatments for emotional disorders at 1-year follow-up [15]. These findings of effect durability are also consistent with the findings of a meta-analysis of long-term outcomes for youth CBTs targeting anxiety [77]; and extend to long-term effect durability for youth CBTs targeting traumatic stress and depression as well [41].

Although our treatment was only six sessions, out of which only two sessions was specific anxiety treatment, our findings are comparable to the results from the Coping Cat study, conducted in CAMHS in Norway, on the CGAS and MASC, although «Coping Cat» is a 12-session treatment solely focusing on anxiety [78]. The results for the present study are also comparable to those found at 6-month follow-up after CBT for depression, as measured with the BDI (e.g. [79]). In a recent meta-analysis of CBT for emotional disorders in routine care number of weeks or sessions did not affect ES or remission rate [18]. Hence, shorter treatments can potentially be as effective as lengthier ones in reducing symptoms of emotional disorders.

Concerning change from baseline to follow-up for various diagnostic groups (no anxiety or depression diagnosis; anxiety; depression; anxiety and depression), the results showed that the groups shared similar patterns of change with reductions of emotional symptoms. The effects are comparable to similar studies on CBT targeting anxiety, depression and trauma [11–13] and meta-analysis of effects found at 1-year follow-up (e.g. [46]). Given that the relapse is usually as high as one third in youths [44, 45], the transdiagnostic treatment of only six sessions of SMART shows promising results also at 6-month follow-up. Although 40% of the sample had 4 or more additional sessions, there was no association between the number of sessions of additional treatment and change in the dependent variables from post-treatment to follow-up.

There were no significant differences for any of the outcome variables in longitudinal trajectories from baseline to follow-up for the two treatment conditions. The two groups received their treatment during different stages of the study, where the treatment group were treated immediately after baseline, and participants in the waiting list condition waited 6 weeks before initiation of the treatment. Hence, there were no indications for different change rate due to differing time schedules in the two groups after therapy had commenced. This implicates that waiting 6 weeks to receive treatment did not have any negative effect when measuring anxiety and depression approximately 6 months after treatment. In general, waiting for treatment has been regarded as negative and great effort has been taken in reducing waiting time [80]. Previous research on physical health services has shown that waiting time is associated with poorer functioning both socially and physical, lower quality of life and poorer health status [81–83]. However, little is known about whether other young patients react negatively to waiting, for instance by reduced attendance, or by developing a more treatment resistant condition while waiting [49]. In this study, the adolescents said yes to this particular treatment, and was told both time frame for waiting and treatment. This would make the situation not just predictable, but would make it possible for them to read about CBT. On the ethical side, it needs to be mentioned that in this study adolescents with the most serious problems had to be excluded from participation due to Governmental restrictions on waiting time for this patient group. Also, 6-week waiting time is relatively short. We have no data to show whether a longer waiting time would have negative effect on long-term outcome.

### Strengths and limitations

The strengths of the present study were that it was performed in a routine clinical setting, that the therapists were representative for ordinary CAMHS, and that the patients were routinely referred cases. Furthermore, a large part of the sample showed comorbid presentations of emotional symptoms, and a variety of symptom measures with good psychometric properties were used, and the interrater reliability of the CGAS, blindly scored by at least three clinicians, was also high. Although the study results has high external validity based on routine care data, limitations concerning the internal validity must be taken into account, since there were high rates of attrition at follow-up, leading to small diagnostic subgroups. This study was not designed with the purpose of comparing the development in different diagnostic groups, which may leave the tests of time by diagnostic group interactions with low power. Therefore, the results have to be interpreted with caution. The linear mixed model analysis applied used information from all 145 subjects when estimating effects. This analysis require that missing data are missing at random (MAR). Sixty-two cases could not be included in the follow-up assessment, and if missing observations are not missing at random (NMAR), estimates may be biased. We do not know if missing data are MAR or not. However, 42 of these cases have been classified as dropout due to “administrative challenges” that consisted of lack of monitoring and clinical routines in conducting follow-up assessment, or sick leave at the clinical administration and not by the patients, and that may reduce the probability of NMAR bias.

The primary outcome measure in this study was the SDQ emotional symptoms scale. Since the patients were screened and included into the study based on their above cut-off score on the SDQ emotional scale, all had homogenously elevated scores at inclusion. One would therefore expect more average change on this measure at follow-up, simply because one would expect that part of the change would be regression to the mean. Similarly, since all patients, baring one, had a daily functioning score (CGAS) indicating being in need of treatment at inclusion, one would also expect that the long-term improvement on this measure was partly caused by regression to the mean. However, for both SDQ emotional symptoms and CGAS the long-term effect sizes were high (d = 2.10 and d = 2.19, respectively), and double the effect sizes found for the secondary outcome measures. Hence, the changes on these two outcome measures were probably too large to solely be explained by regression to the mean. However, the standard deviation for SDQ emotional scale was low because of the screening of the patients and large effect sizes on this scale could also be explained by low standard deviations. On the other hand, the inclusion into the study was not based on the scores on measures of general psychological distress (CORE-17), depression (BDI-II) and anxiety (MASC). Thus, the distributions at inclusion for those outcome variables were more heterogeneous, and those scoring in the lower end of the distribution would have little chance for systematic improvement on these scales, due to so called floor effects. Nevertheless, the observed effect sizes at follow-up were quite large, also for these three measures.

This study supports the growing evidence that transdiagnostic treatments are effective in treating depression and anxiety [84]. Furthermore, the results support a broad and pragmatic clinical approach to treating internalization disorders in youths; with offering a short-term transdiagnostic treatment as the first choice, and a more tailored and diagnosis specific treatment as the second choice, if needed. The study should be replicated with an active control group also at follow-up to rule out possible placebo effects and to evaluate what incremental effects the SMART treatment can contribute compared to treatment already established in the CAMHS.

## Conclusions

Six weeks of transdiagnostic treatment with the SMART program for emotional problems showed promising results with large significant change in overall emotional symptoms and significant improvement in daily functioning in a follow-up at 6 months post-treatment. There were no significant treatment group or diagnostic group differences in the overall rate of change from baseline to follow-up in the non-diagnosis group, depression only, anxiety only, and depression and anxiety combined group. If a six session transdiagnostic treatment can be acceptable, and have lasting impact, it is a scalable and likely cost-effective treatment to be considered as the first step in a stepped care model in CAMHS for youths with emotional disorders. The results also illuminate the need for further treatment for some of the patients.

## Data Availability

The datasets generated and analyzed during the current study are available from the corresponding author on reasonable request.

## Abbreviations

CBT: Cognitive behavioral therapy
CAMHS: Children and adolescent mental health services
WLC: Wait list control
SDQ: Strength and difficulties questionnaire
SD: Standard deviation
EST: Empirically supported treatment
ES: Effect size
UP-A: Unified protocol for the treatment of emotional disorders
RCT: Randomized controlled trial
SMART: Structured material for therapy
PDD: Pervasive developmental disorder
DAWBA: Development and well being assessment
CGAS: The children's global assessment scale
CORE-OM: Clinical outcome in routine evaluation-outcome measure
BDI-II: Beck depression inventory, second edition
MASC: Multidimensional anxiety scale for children
